# MiR‐15a/16‐1 deficiency induces IL‐10‐producing CD19^+^ TIM‐1^+^ cells in tumor microenvironment

**DOI:** 10.1111/jcmm.14037

**Published:** 2018-11-23

**Authors:** Xiaoqin Jia, Hao Liu, Chong Xu, Sen Han, Yating Shen, Xin Miao, Xiangyu Hu, Zhijie Lin, Li Qian, Zhengbing Wang, Weijuan Gong

**Affiliations:** ^1^ Department of Basic Medicine, Institute of Translational Medicine, Medical College Yangzhou University Yangzhou P.R. China; ^2^ Jiangsu Key Laboratory of Experimental & Translational Non‐coding RNA Research Yangzhou P.R. China; ^3^ Department of General Surgery Subei People’s Hospital of Jiangsu Province, Yangzhou University Yangzhou P.R. China; ^4^ Jiangsu Key Laboratory of Integrated Traditional Chinese and Western Medicine for Prevention and Treatment of Senile Diseases Yangzhou P.R. China; ^5^ Jiangsu Key Laboratory of Zoonosis Yangzhou P.R. China; ^6^ Jiangsu Co‐innovation Center for Prevention and Control of Important Animal Infectious Diseases and Zoonoses Yangzhou P.R. China

**Keywords:** CD19, IL‐10, miR‐15a/16, Tim‐1, tumor

## Abstract

IL‐10‐producing B cells (B10) are associated with autoimmune diseases, infection and tumours. MiR‐15a/16 as a tumour‐suppressive gene is down‐regulated in several tumours, such as chronic lymphocytic leukaemia, pituitary adenomas and prostate carcinoma. Here, increased frequency of IL‐10‐producing CD19^+^ Tim‐1^+^ cells was seen in both aged miR‐15a/16^−/−^ mice (15‐18 months) with the onset of B cell leukaemia and young knockout mice (8‐12 weeks) transplanted with hepatic cancer cells. CD19^+^ Tim‐1^+^ cells down‐regulated the function of effector CD4^+^CD25^low^ T cells ex vivo dependent on IL‐10 production, and adoptive transfer of CD19^+^ Tim‐1^+^ cells promoted tumour growth in mice. IL‐10 production by CD19^+^ Tim‐1^+^ cells was involved with the STAT3 activation. Bioinformatics analysis shows that miR‐16 targets the 3′‐untranslating region (3′‐UTR) of STAT3 mRNA. Overexpression of miR‐16 in CD19^+^ Tim‐1^+^ cells inhibited STAT3 transcription and its protein expression. Thus, the loss of miR‐15a/16 promoted induction of regulatory CD19^+^ Tim‐1^+^ cells in tumour microenvironment. These results confirmed that miR‐15a/16 could be used in tumour therapy due to its inhibition of tumour and regulatory B cells.

## 1 INTRODUCTION

The IL‐10‐secreting B cells (B10), as one of the important regulatory B (Breg) cells, are associated with autoimmune diseases, infections and tumours. B10 cells can directly inhibit macrophage activation, down‐regulate its secretion of NO and related inflammatory cytokines (TNF‐α, IL‐1, IL‐6, IL‐8, IL‐12), and also reduce the phagocytosis of macrophages.^1^, ^2^ Breg cells can inhibit antigen presentation and expression of costimulatory molecules and cytokines of dendritic cells (DCs). The Th1/Th17 effector cells are also regulated by Breg cells to down‐regulate the local inflammatory response. In addition, Breg cells can also induce classical Treg and Tr1 cells to promote immune‐regulatory activity.^3^, ^4^

So far, three subsets of IL‐10‐secreting B cells have been identified, namely, CD19^+^ CD5^+^ CD1d^hi^,^5^, ^6^ CD19^+^ Tim^+^ 7 and CD19^+^FcγIIb^hi^ 8 cells. After being stimulated with Tim‐1 antibody, 45% of CD19^+^CD5^+^CD1d^hi^ cells up‐regulated Tim‐1 expression and secreted IL‐10, whereas 8% of them could express Tim‐1 and had potential IL‐10 secretion ability in non‐CD5^+^ CD1d^hi^ B cells, which suggested that these two phenotypic Breg cells could not replace each other.^7^ A unique phenotype of CD19^hi^FcγIIb^hi^ B cells, which is induced by regulatory DCs, also produces IL‐10 to inhibit effector CD4^+^ T cells.^8^ The engagement of BCR is thought to play a role in the early stage, whereas the activation of CD40 and Toll‐like receptors (TLRs) plays a role in the late stage of Breg cell differentiation. Several signalling proteins, such as Erk, p38, glycogen synthase kinase 3‐β (GSK3‐β), Smad, Signal transducers and activators of transcription (STATs), mammalian target of rapamycin (mTOR) and nuclear factor‐kappa B (NF‐κB), are involved in IL‐10 production.^9^, ^10^, ^11^

The miR‐15a/16 gene complex is located in the intron of the DLEU2 gene in the human 13q14 region, which is recognized as a tumour‐suppressor gene.^12^ The deletion of this gene region is closely related not only with chronic B lymphocytic leukaemia, but also with various solid tumours such as melanoma, colorectal cancer, prostate cancer, breast cancer and bladder cancer.^13^ The target genes of miR‐15a/16 include Bcl‐2, WT‐1, WNT3A, MCA1, MCL1 and CCDN1, which are involved in tumour apoptosis.^14^ MiR‐15a/16 can also inhibit vascular endothelial growth factor (VEGF) secretion and play anti‐angiogenesis activity.^15^ Here, we showed that the deficiency of miR‐15a/16 was associated with induction of IL‐10‐producing Breg cells under tumour microenvironment by up‐regulating STAT3 expression.

## 2 MATERIALS AND METHODS

### 2.1 Reagents and mice

MiR‐15a/16^−/−^ mice (C57BL/6) were obtained from the Jackson Laboratory (Sacramento, CA). All animal experiments were approved by the Institutional Animal Care and Use Committee of Yangzhou University (Yangzhou, China). The murine liver cancer cell line (H22) was obtained from ATCC. Antibodies for flow cytometry were purchased from either BioLegend (San Diego, CA) or eBioscience (San Diego, CA): CD19 (1D3), B220 (RA3‐6B2), CD5 (53‐7.3), Tim‐1 (RMT1‐4), FcγIIb (AT130‐2), IL‐10 (JES5‐16E3), CD1d (1B1), CD4 (RM4‐5), CD69 (H1.2F3) and IFN‐γ (XMG1.2). Antibodies for Western blot were from Cell Signaling Technology (Boston, MA): STAT3 (4904P), STAT3‐pY705 (9145S) and STAT3‐pS727 (94994T).

### 2.2 ELISA

Splenic B cells (5 × 10^5^) isolated either from miR‐15a/16^−/−^ or wild‐type (WT) mice (C57BL/6) by magnetic sorting beads (Miltenyi) were cultured in complete RPMI 1640 medium. For 24 or 48 hours, cell supernatants were collected, and IL‐10 concentrations were measured according to the manufacturer's protocol (BioLegend). Serum IL‐10 levels of miR‐15a/16 knock‐out (KO) or wild‐type (WT) mice were also determined by the same kit.

### 2.3 Flow cytometric intracellular staining

Intracellular cytokine production was determined using a staining kit (eBioscience). For IL‐10 detection, B lymphocytes were cultured in the presence of Brefeldin A (10 μg/mL) for 4 hours at 37°C. For IFN‐γ measurement, lymphocytes were stimulated with PMA (50 ng/mL)/ionomycin (5 μg/mL) in the presence of Brefeldin A for 4 hours at 37°C. After lymphocytes were stained with surface markers, they were fixed, permeabilized, stained with cytokine or isotype antibody and analysed by flow cytometry.

### 2.4 Coculture of B and T cells

CD19^+^ Tim‐1^+^ cells were isolated from spleens of WT or KO mice which were pre‐transplanted with H22 cells by flow cytometry. CD4^+^CD25^high^ or CD4^+^CD25^low^ cells from normal mice (C57BL/6) were also sorted out by flow cytometry. Then, two lymphocyte subsets were mixed in a 1:1 ratio and cultured overnight. The mixed lymphocytes were collected, and CD69 expression and IFN‐γ secretion were analysed in CD4^+^CD25^high^ or CD4^+^CD25^low^ cells as described above.

### 2.5 In vivo mouse tumour models

When H22 cells (2 × 10^6^) were subcutaneously injected into mice (n = 3), CD19^+^ Tim‐1^+^ or CD19^+^ Tim‐1^−^ cells (5 × 10^5^) sorted form KO mice were injected into the tail veins of tumour‐bearing mice every day. Tumour diameters were also documented every day. On day 30, mice were sacrificed and tumour tissues were isolated.

### 2.6 Western blot

Proteins of CD19^+^ Tim‐1^+^ cells either from miR‐15a/16^−/−^ or WT mice were extracted after the lysis buffer (KeyGen, Nanjing, China) was added into cells. After being separated on SDS‐PAGE gels and transferred onto polyvinylidene difluoride (PVDF) membranes, proteins were stained with first and secondary antibodies sequentially. The blotting signal was developed using an ECL kit (KeyGen) and analysed with the Gel‐Pro32 software.

### 2.7 Lentivirus infection

Lentivirus‐expressing miR‐16‐1 (LV‐miR‐16) and a control lentivirus (LV‐control) were provided by GeneChem (Shanghai, China). The transfection procedure was the same as in the previous study.^16^

### 2.8 Real‐time polymerase chain reaction (PCR)

As CD19^+^ Tim‐1^+^ cells from miR‐15a/16^−/−^ mice were transfected with the LV‐miR‐16 lentivirus for 72 hours, RNA was extracted by the TRIzol reagent (Life Technologies; Carlsbad, CA), and cDNA was generated by a QuantiTect^®^ reverse transcription kit (QIAGEN GmbH; Hilden, Germany). The amplification of cDNA was conducted using the QuantiNova™ SYBR^®^ Green PCR Kit (QIAGEN) on ABI 7500 (PE Applied Biosystems, Carlsbad, CA, USA). Primer pairs for STAT3 were 5′‐CACCCAACAGCCGCCGTAGT and 5′‐TCCATTCAGATCCTGCATGTCTCC.

### 2.9 Statistical analysis

Differences between the two groups were analysed by Student's *t* test. Data were evaluated by one‐way ANOVA followed by Dunnett's test between control and various stimulation groups. Significant differences were indicated when *P < 0.05, **P < 0.01, and ***P < 0.001.

## 3 RESULTS

### 3.1 Increased IL‐10‐producing B cells in the aged knockout (KO) mice with leukaemia

**Figure 1:**
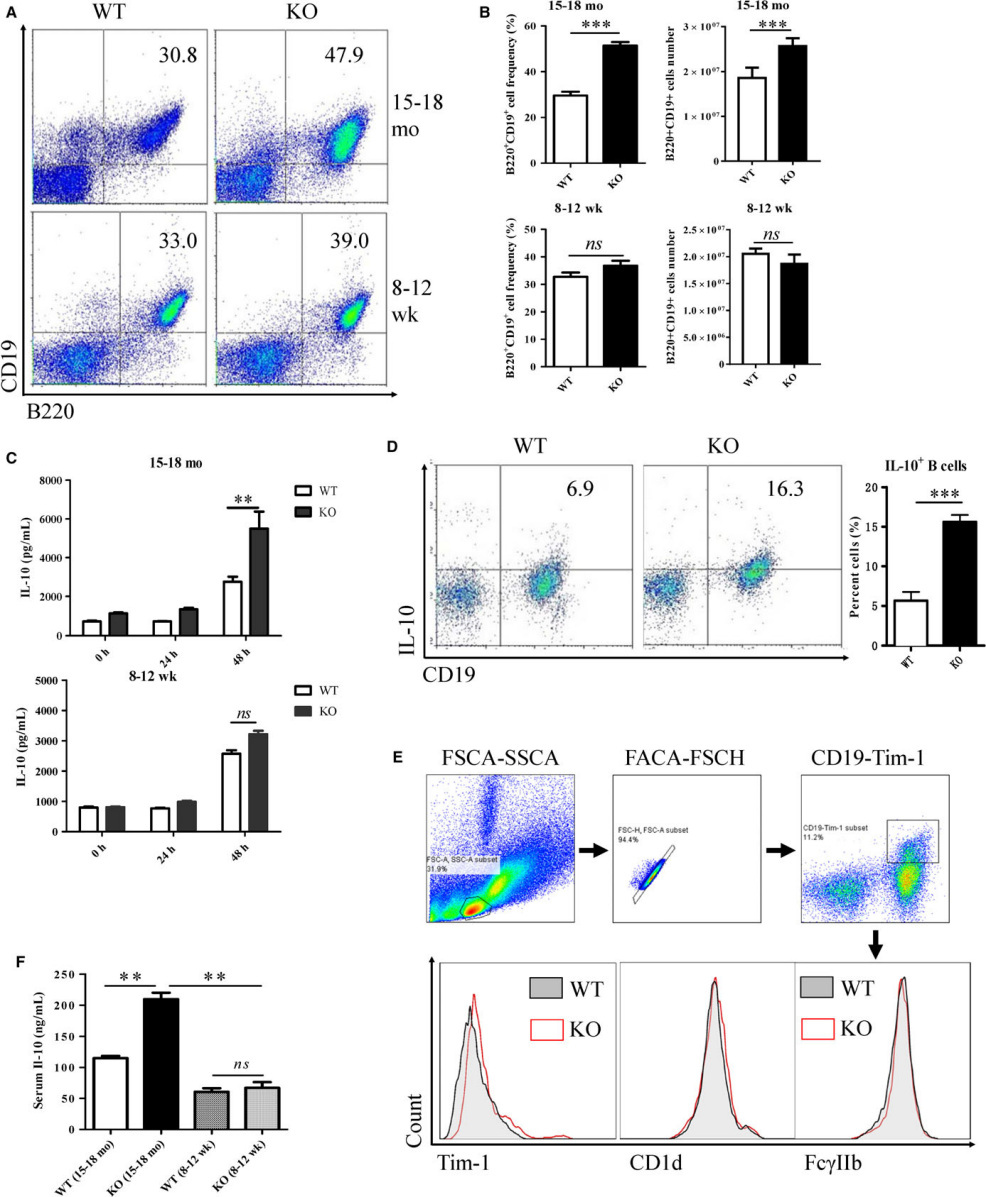
Enhanced IL‐10‐producing B cells in the aged (15‐18 months) KO mice. (A, B) Detection of CD19^+^ B220^+^ cells of spleens from both aged and young (8‐12 weeks) mice by flow cytometry. (C) After CD19^+^ cells were isolated by magnetic beads and cultured ex vivo, cell supernatants were collected, and IL‐10 concentrations were measured with an ELISA kit. (D) Intracellular IL‐10 of B cells from the aged mice was detected by flow cytometry. (E) Expression of Tim‐1, CD1d and FcγIIb on CD19^+ ^IL‐10^+^cells detected by flow cytometry after multicolour fluorescence labelling. (F) Serum IL‐10 levels of aged and young KO or WT mice. **P < 0.01; ***P < 0.001; ns, no significance.

The miR‐15a/16^−/−^ mice spontaneously develop B cell leukaemia at the age of 15‐18 months with a penetrance of 60%.^17^ First, B cell frequencies in spleens of KO or WT mice in the age of 15‐18 months or 8‐12 weeks were analysed. The aged KO mice (15‐18 months) were verified to have B cell leukaemia as shown in the Figure S1. As expected, B cell frequency and absolute number are significantly enhanced in the aged KO mice (15‐18 months) (Figure 1A,B). No changes of B cells were observed in young KO mice (8‐12 weeks), compared with WT mice. B cells from spleens of KO or WT mice at different ages were isolated and cultured ex vivo for 24 or 48 hours. In aged mice, IL‐10 concentrations of supernatants from KO mice‐derived B cells were significantly higher than those from WT mice‐derived B cells. We did not observe any significant differences of IL‐10 concentration of B cell supernatants from young mice (Figure 1C). IL‐10 production by B cells from aged KO mice was confirmed by intracellular staining of flow cytometry, as shown in Figure 1D. Multi‐colour fluorescence labelling was used to analyse surface markers of CD19^+^IL‐10^+^ cells. KO mice‐derived CD19^+^IL‐10^+^ cells displayed the increased expression of Tim‐1 and the similar expression of CD1d and FcγIIb, as compared with WT mice (Figure 1E). Serum IL‐10 level of aged KO mice was also higher than that of aged WT mice and young KO mice (Figure 1F). Thus, there was a B cell population with IL‐10‐producing activity in the aged KO mice.

### 3.2 Increased CD19^+^ Tim‐1^+^ cells in the aged KO mice with leukaemia

**Figure 2:**
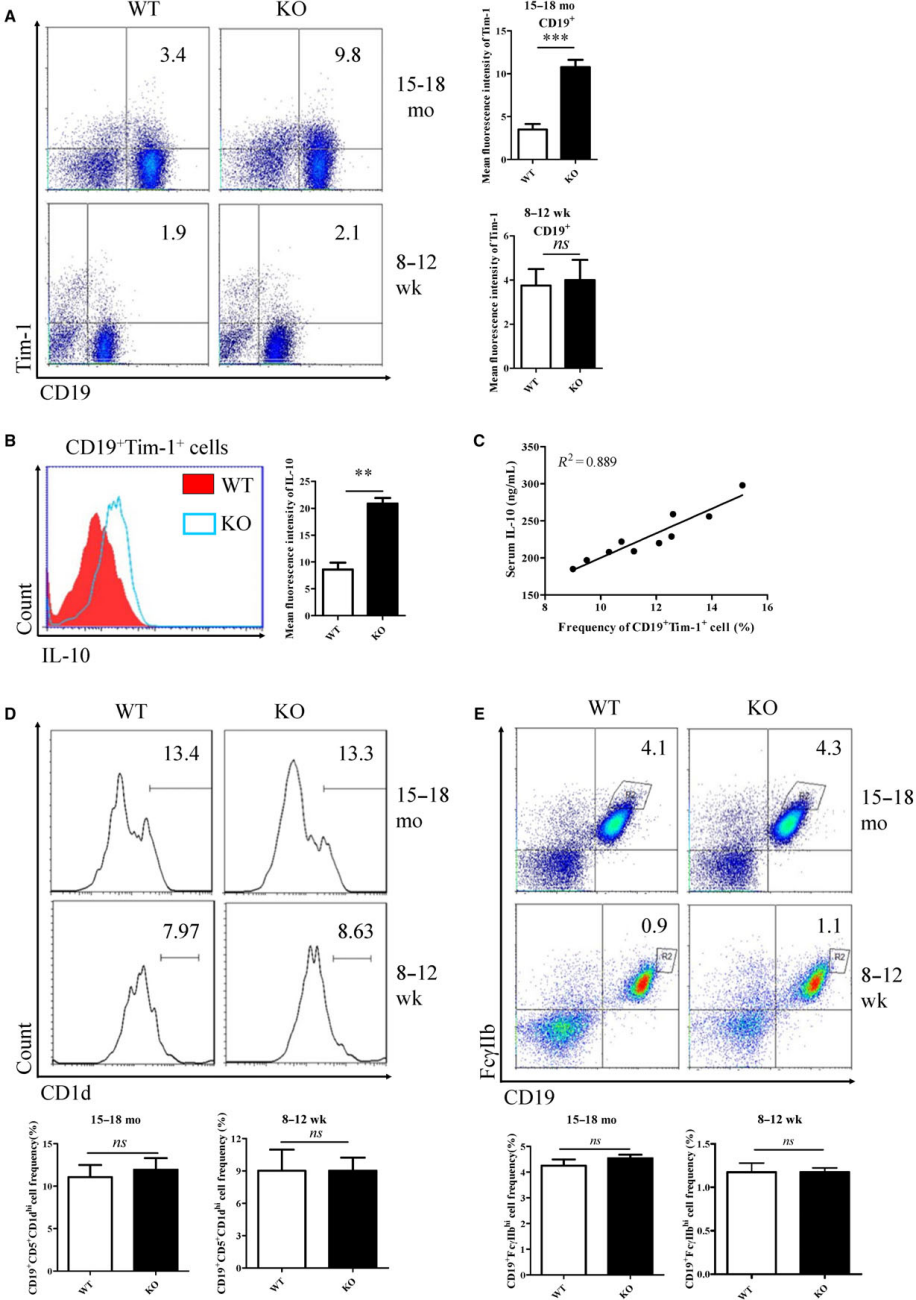
Analysis of regulatory B cell subsets in the aged (15‐18 months) KO mice. CD19^+^Tim‐1^+^ (A), CD19^+^CD5^+^CD1d^hi^ (D), and CD19^+^FcγIIb^hi^ (E) cell frequencies in spleens were detected by flow cytometry. (B) IL‐10 production by CD19^+^Tim‐1^+^ were determined by intracellular staining of flow cytometry. (C) Correlation analysis of CD19^+^Tim‐1^+^ cell frequency with serum IL‐10. **P < 0.01; ns, no significance

Three Breg cell populations with IL‐10 production (CD19^+^ Tim‐1^+^, CD19^+^CD5^+^CD1d^hi^, CD19^+^FcγIIb^hi^) were detected in both aged and young KO mice. In the aged KO mice (15‐18 months), splenic CD19^+^ Tim‐1^+^ cell frequency was significantly increased compared with WT mice at the same age. No significant changes of splenic CD19^+^ Tim‐1^+^ cell frequency were observed in young mice (Figure 2A). These CD19^+^ Tim‐1^+^ cells were able to secrete IL‐10 as detected by flow cytometry (Figure 2B). The frequency of CD19^+^ Tim‐1^+^ cells was positively correlated with serum IL‐10 level (Figure 2C). However, the CD19^+^CD5^+^CD1d^hi^ cell frequency did not vary obviously in aged or young KO mice (Figure 2D). CD19^+ ^FcγIIb^hi^ cells also showed no significant changes between KO and WT mice at different ages (Figure 2E). Therefore, CD19^+^ Tim‐1^+^ cells with IL‐10 production were induced in aged KO mice bearing B cell leukaemia.

### 3.3 Increased CD19^+^ Tim‐1^+^ cells in young KO mice pre‐transplanted with tumour cells

**Figure 3:**
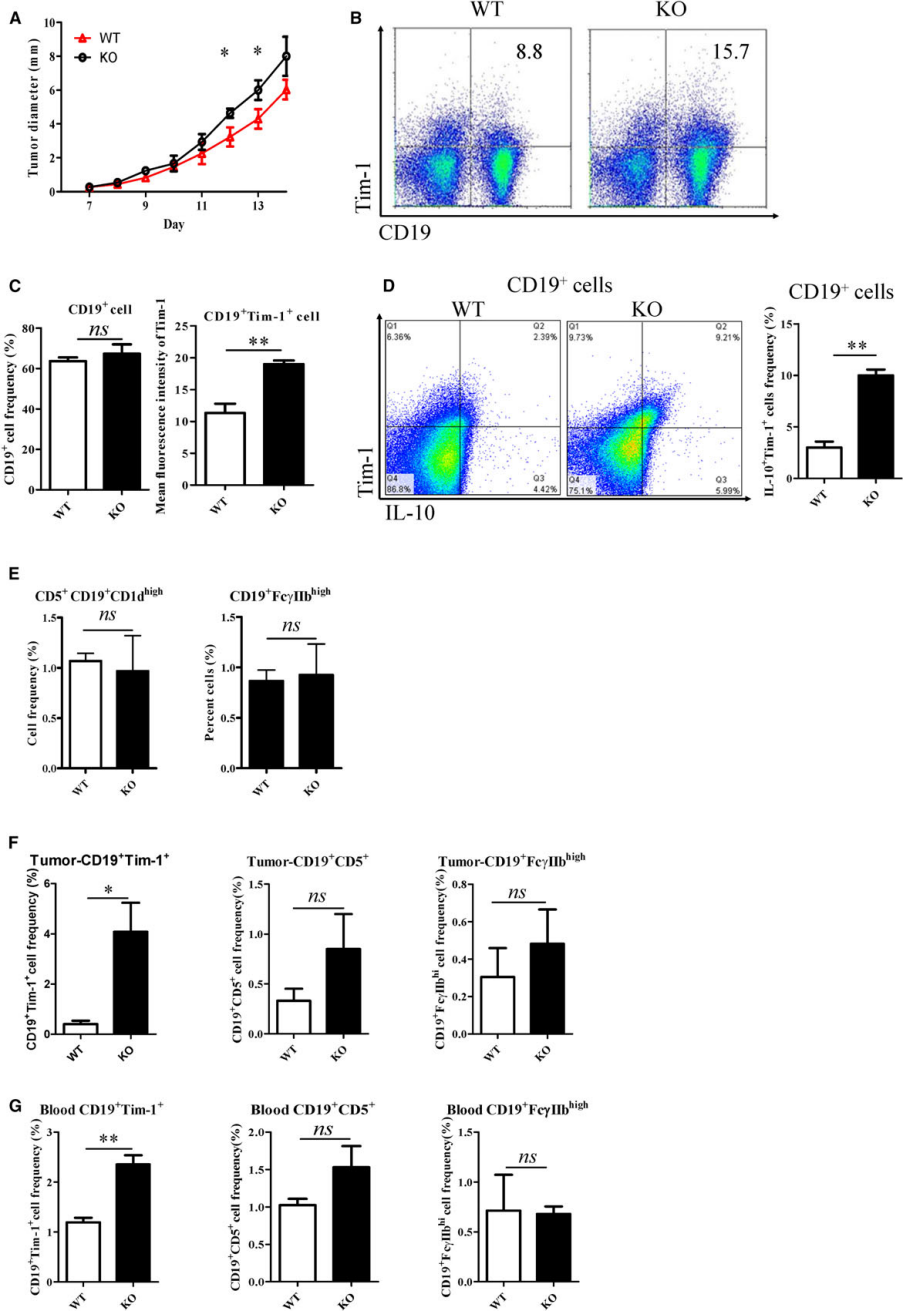
Analysis of Breg cell subsets in young (8‐12 weeks) KO mice bearing with transplanted tumours. (A) Tumour growth curve in KO and WT mice after H‐22 cells (5 × 10^6^) were subcutaneously injected. (B, C) CD19^+^ Tim‐1^+ ^cell frequencies in spleens were detected by flow cytometry. (D) Il‐10^+^ Tim‐1^+^ cells frequency gated on CD19^+^ cell was determined by intracellular staining of flow cytometry. (E) Splenic CD19^+^CD5^+^CD1d^hi^ and CD19^+^FcγIIb^hi^ cell frequencies of tumour‐bearing mice. CD19^+^ Tim‐1^+^, CD19^+ ^CD5^+^ and CD19^+ ^FcγIIb^hi^ cell frequencies of tumour tissues (F) and peripheral blood (G). *P < 0.05; ns, no significance

Considering IL‐10‐secreting B cells and leukaemic B cells in the aged B cell‐leukaemia bearing miR‐15a/16^−/−^ mice (15‐18 months) could not be discriminated clearly, we determined whether CD19^+^ Tim‐1^+^ cells could be induced in young KO mice (8‐12 weeks) bearing with solid tumours. Hepatic cancer cells (H22) were subcutaneously transplanted into back of KO or WT mice. Tumour growth was up‐regulated in KO mice indicated by tumour size (Figure 3A). After 14 days of H22 transplantation, Breg cells of KO or WT mice were analysed. Compared with that in tumour‐bearing WT mice, the splenic CD19^+^ Tim‐1^+^ cell frequency was significantly increased in tumour‐bearing KO mice (Figure 3B,C). Frequency of CD19^+ ^IL‐10^+^ Tim‐1^+^ cells was also increased in KO mice (Figure 3D). Similarly, no significant changes of splenic CD19^+^CD5^+^CD1d^hi^ and CD19^+^FcγIIb^hi^ cells were observed in the two groups of mice (Figure 3E). The representative results of CD19^+^CD5^+^CD1d^hi^ and CD19^+^FcγIIb^hi^ cells detected by flow cytometry were shown in Figure S2. Simultaneously, CD19^+^ Tim‐1^+^ cell frequencies in tumour tissues (Figure 3F) and peripheral blood (Figure 3G) were significantly increased in KO mice. Because sufficient CD19^+^CD5^+ ^cells infiltrated in tumour tissues can't be acquired for analysis of distinct CD1d expression levels, we only quantified the frequency of CD19^+^CD5^+ ^cells in tumour tissues and peripheral blood (Figure S3). As shown in Figures 3F,G, there were no significant differences of CD19^+^ CD5^+^ and CD19^+ ^FcγIIb^hi^ cell frequencies in tumour tissues and peripheral blood between two strains of mice. Therefore, CD19^+^ Tim‐1^+^ cells with IL‐10 production could be also induced in young KO mice transplanted with tumour cells.

### 3.4 Regulatory activity of CD19^+^ Tim‐1^+^ cells depends on IL‐10 production

**Figure 4:**
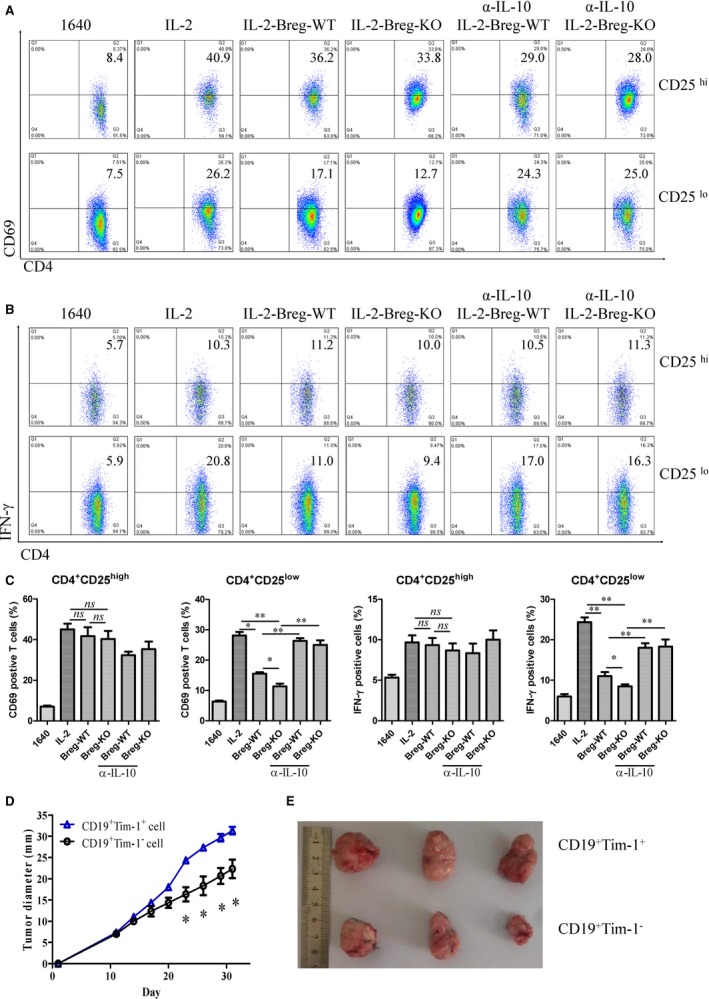
Immune‐regulatory function mediated by CD19^+^ Tim‐1^+^ cells via IL‐10 production. (A, C) Variations of CD69 expression of IL‐2‐stimulated CD4^+^ T cells from healthy C57BL/6 mice after in coculture with CD19^+^ Tim‐1^+^ cells at a ratio of 1:1 (with/without IL‐10 neutralizing antibody, 10 μg/mL). (B, C) IFN‐γ production by CD4^+^ T cells was detected by intracellular staining of flow cytometry after being incubated with CD19^+^ Tim‐1^+^ cells. (D) Growth curve of transplanted H22 cells after adoptive transfer of CD19^+^ Tim‐1^+^ or CD19^+^ Tim‐1^−^ cells. (E) Stripped tumour tissues from mice. Each experiment was repeated at least thrice. *P < 0.05; **P < 0.01; ***P < 0.001

CD4^+ ^CD25^high^ cells are generally regarded as regulatory T cells, and CD4^+^ CD25^low^ cells as effector T cells. When CD19^+^ Tim‐1^+^ cells were incubated with IL‐2‐stimulating CD4^+^ cells, CD69 expression was significantly down‐regulated on CD4^+ ^CD25^low^ cells, but no changes on CD4^+^ CD25^high^ cells (Figure 4A,C). Moreover, IFN‐γ production was sharply inhibited in CD4^+^ CD25^low^ cells after these T cells were cocultured with CD19^+ ^Tim‐1^+^ cells (Figure 4B,C). The suppressive activity of Tim‐1^+^ B‐cells from KO mice was also higher than that of WT mice (Figure 4B,C). Thus, CD19^+^ Tim‐1^+^ cells exerted immune‐regulatory effects through inhibiting activity of effector T cells. When IL‐10 neutralizing antibodies were added into the B‐T cell coculture system, the inhibitory effect of CD69 and IFN‐γ expression was almost reversed (Figure 4A‐C), demonstrating that Breg cells mediated the regulatory effect via IL‐10 secretion. Next, CD19^+^ Tim‐1^+^ or CD19^+^ Tim‐1^−^ cells from KO mice were adoptively transferred into mice pre‐injected with H22 cells every 3 days and lasted for 30 days. As expected, the transfer of CD19^+^ Tim‐1^+^ cells significantly promoted tumour growth in vivo (Figure 4D,E).

### 3.5 STAT3 contributes to IL‐10 production of CD19^+^ Tim‐1^+^ cells

**Figure 5:**
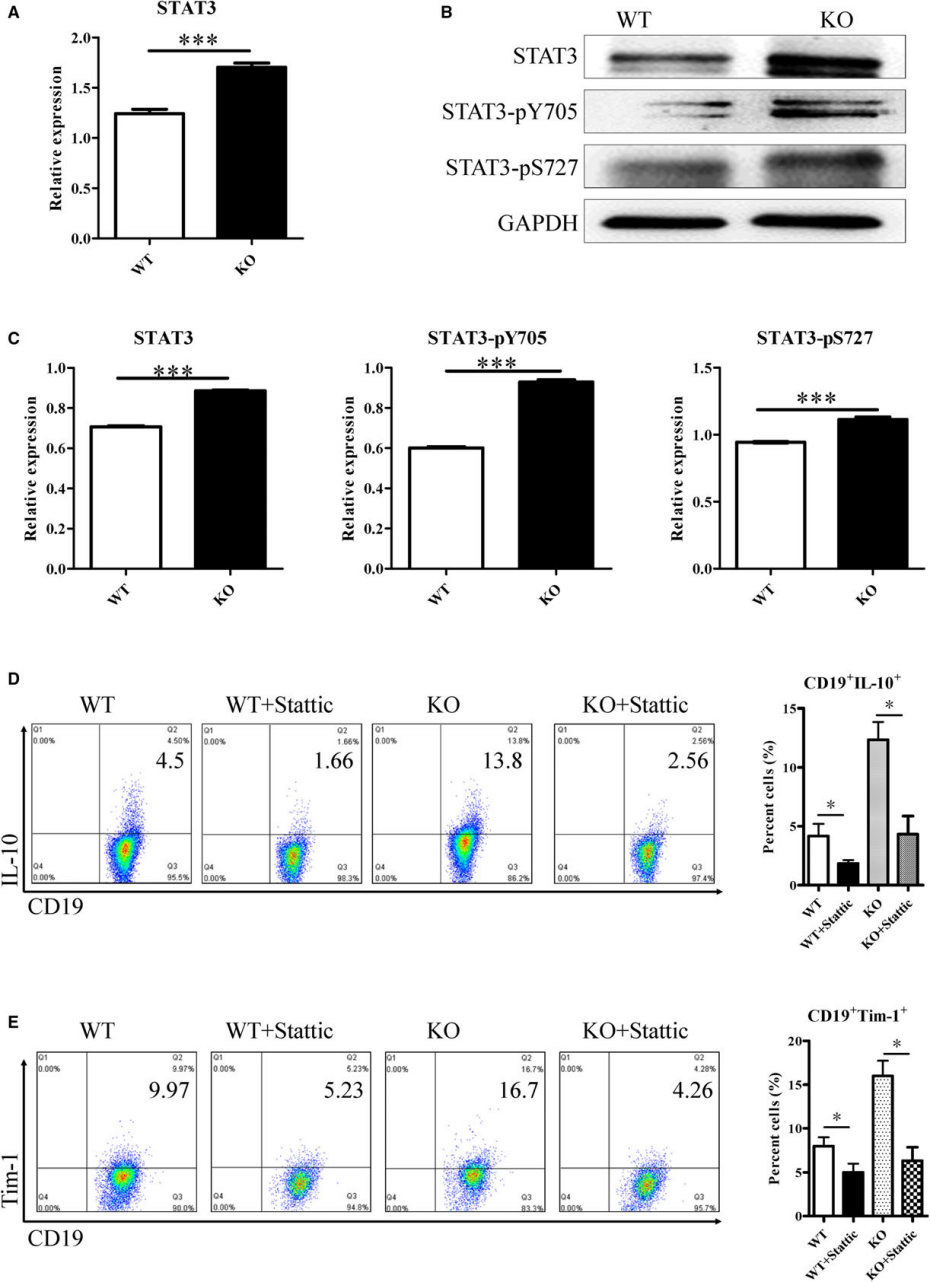
STAT3 activity contributes to IL‐10 production by CD19^+^ Tim‐1^+^ cells. (A) STAT3 mRNAs of CD19^+^ Tim‐1^+^ cells from both mice were measured by reverse transcription and real‐time PCR. (B) Total STAT3, STAT3‐pY705, and STAT3‐pS727 levels were determined by Western blot. (C) Statistical analysis of STAT3 expression in CD19^+^ Tim‐1^+^ cells from both mice. Variations of IL‐10 (D) and Tim‐1 (E) expression in CD19^+^ Tim‐1^+^ cells treated with the STAT3 inhibitor (Stattic). Each experiment was repeated at least thrice. *P < 0.05; ***P < 0.001

STAT3 activation is involved in IL‐10 production of immune cells. STAT3 levels of CD19^+^ Tim‐1^+^ cells were checked in tumour‐bearing KO and WT mice. STAT3 mRNA levels were higher in CD19^+^ Tim‐1^+^ cells of KO mice than those of WT mice (Figure 5A). Total STAT3, STAT3‐pY705 and STAT3‐pS727 were all increased in CD19^+^ Tim‐1^+^ cells of tumour‐bearing KO mice (Figure 5B,C). When the STAT3 inhibitor (Stattic) was treated with KO mice‐derived CD19^+^ Tim‐1^+^ cells, IL‐10 production and Tim‐1 expression are both blocked (Figure 5D,E). The result indicated that the increased STAT3 activity contributed to IL‐10 production by CD19^+^ Tim‐1^+^ cells.

### 3.6 MiR‐16 over‐expression in CD19^+^ Tim‐1^+^ cells inhibits STAT3

**Figure 6:**
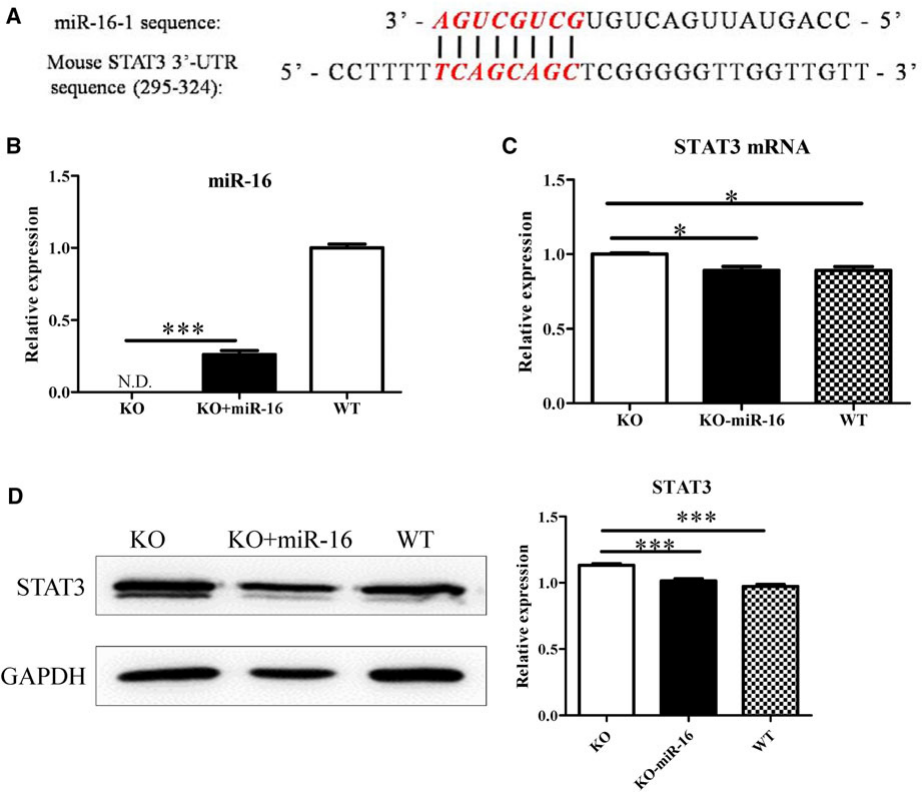
Overexpression of miR‐16 down‐regulates STAT3. (A) Bioinformatics analysis of complementary sequences between miR16 and 3′‐UTR of STAT3 mRNA. (B) miR‐16 expression of CD19^+^Tim‐1^+^ cells determined by RT‐PCR. (C) After CD19^+^ Tim‐1^+^ cells were transfected by the lentivirus containing miR‐16, STAT3 mRNA was detected by RT‐PCR. (D) Variations of STAT3 protein levels in CD19^+^ Tim‐1^+^ cells detected by Western blot after the miR‐16 lentivirus transfection. Each experiment was repeated at least thrice. *P < 0.05; ***P < 0.001

Next, we determined whether miR‐15a/16 directly down‐regulated the STAT3 mRNA level. By using bioinformatics analysis, miR‐16, not miR‐15a, was found to directly bind with the 3′‐untranslated region (UTR) of STAT3 mRNA (Figure 6A). After KO mice‐derived CD19^+^ Tim‐1^+^ cells were transfected with miR‐16‐containing lentivirus, miR‐16 expression levels were verified and shown in Figure 6B. At the same time, the transcription of STAT3 was significantly inhibited (Figure 6C). Western blot results also confirmed that STAT3 was down‐regulated in CD19^+^ Tim‐1^+^ cells by being transfected with the miR‐16‐containing lentivirus (Figure 6D). Therefore, overexpression of miR‐16 in KO mice‐derived CD19^+^ Tim‐1^+^ cells suppressed STAT3 expression.

## 4 DISCUSSION

The microRNA cluster of miR‐15a/miR‐16‐1 (miR‐15a/16) located at 13q14.2 of the chromosome is regarded as a tumour‐suppressive gene. The loss of the gene cluster is involved in the development of cancers such as chronic lymphocytic leukaemia (CLL), pituitary adenomas, and prostate carcinoma.^13^, ^14^ Here, we determined that the loss of miR‐15a/16 was associated with induction of IL‐10‐producing B cells under tumour microenvironment. Breg cells could down‐regulate biologic activities of effector CD4^+^ T cells and promote tumour growth with the characteristic phenotype of CD19^+^ Tim^+^. IL‐10 production by B cells was dependent on the STAT3 activation, and overexpression of miR‐16 resulted in inhibition of STAT3 and suppression of CD19^+^Tim‐1^+^ cells. We described that Tim‐1^+^ Breg cells with immune‐suppressive activity for tumour evasion was involved with the loss of the miR‐15a/16 gene cluster.

In humans, malignant cells from 90% of patients with CLL are able to produce IL‐10. These IL‐10‐competent CLL cells also express cell surface phenotypes similar to nonmalignant B10 cells, indicating a functional relationship between CLL and B10 cells.^18^ We could not discriminate B10 cells and leukemic B cells in the aged miR‐15a/16^−/−^ mice (15‐18 months) bearing B cell leukaemia. When the young miR‐15a/16^−/−^ mice (8‐12 week) were transplanted with hepatic cancer cells, IL‐10‐producing CD19^+^ Tim‐1^+^ cells were significantly increased. In this case, as there is no B cell leukaemia in mice, IL‐10‐producing CD19^+^ Tim‐1^+^ cells were induced instead. No obvious changes in IL‐10‐producing CD19^+^ Tim‐1^+^ cells were observed in healthy young miR‐15a/16^−/−^ mice, suggesting that this Breg subset was only induced in tumour microenvironment. Breg cells could be recruited to the tumour and thereby attenuate anti‐tumour immune responses.

To date, the detailed molecular mechanisms of Breg development in the tumour microenvironment remain unknown. Some tumour cell‐derived factors, such as leukotriene B4,^19^ TNF‐α,^20^ placental growth factor^21^ and IL‐21 secreted by local T cells,^22^ have been recognized as Breg‐induced factors. In addition, cell membrane molecules (CD40L^23^ or PD‐1^24^) of tumours are involved in Breg development. The differentiation of Breg cells mainly depends on the engagement of BCR and CD40.^25^, ^26^ In human B cells, STAT3 and Erk activation induced by TLR controls IL‐10 expression.^27^ The inhibition of STAT3 blocked IL‐10 expression by CD19^+^ Tim‐1^+^ cells, suggesting that using STAT3 inhibitors in tumour patients also retards B10 cell development.

Ectopic expression of miR‐15a and miR‐16‐1 has been shown to up‐regulate 265 genes and down‐regulate 3307 genes.^28^ We found that eight nucleotides of miR‐16 are complementary to bases 295‐324 at the 3′‐end of the STAT3 cDNA. The overexpression of miR‐16 led to the down‐regulation of STAT3 mRNA and protein levels in CD19^+^ Tim‐1^+^ cells from KO mice. Whether miR‐16 regulates STAT3 expression through direct binding of its 3′‐UTR needs further study. It could be inferred that the STAT3 expression regulated by miR‐16 was not as strong as that by other miR‐16 prominent target genes (eg, *BCL‐2*, *MCL1*, *CCND1* and *WNT3A*).^13^, ^14^ In addition, although we analysed the activity of miR‐16 in B10 cell development, the role of miR‐15a (belonging to the same microRNA family) could not be excluded.

In summary, deficiency of the microRNA cluster of miR‐15a/16 promoted the induction of IL‐10‐producing CD19^+^ Tim‐1^+^ cells in mice. The development of regulatory CD19^+^ Tim‐1^+^ cells was dependent on STAT3 activation. Overexpression of miR‐16 inhibited STAT3 expression. Considering that microRNAs target many genes, this study confirmed that miR‐15/16 could be used pharmaceutically in tumour therapy.^29^, ^30^

## CONFLICT OF INTEREST

All authors have declared there are no financial conflicts of interest with regard to this work.
