# Supplementary material for: MiR‐15a/16‐1 deficiency induces IL‐10‐producing CD19+ TIM‐1+ cells in tumor microenvironment
Source: J Cell Mol Med. 2018 Nov 23;23(2):1343–53. doi: 10.1111/jcmm.14037 (PMC6349175; doi:10.1111/jcmm.14037)
Supplement: Supplementary file 1 [file JCMM-23-1343-s001.pdf]

Supplementary Figure

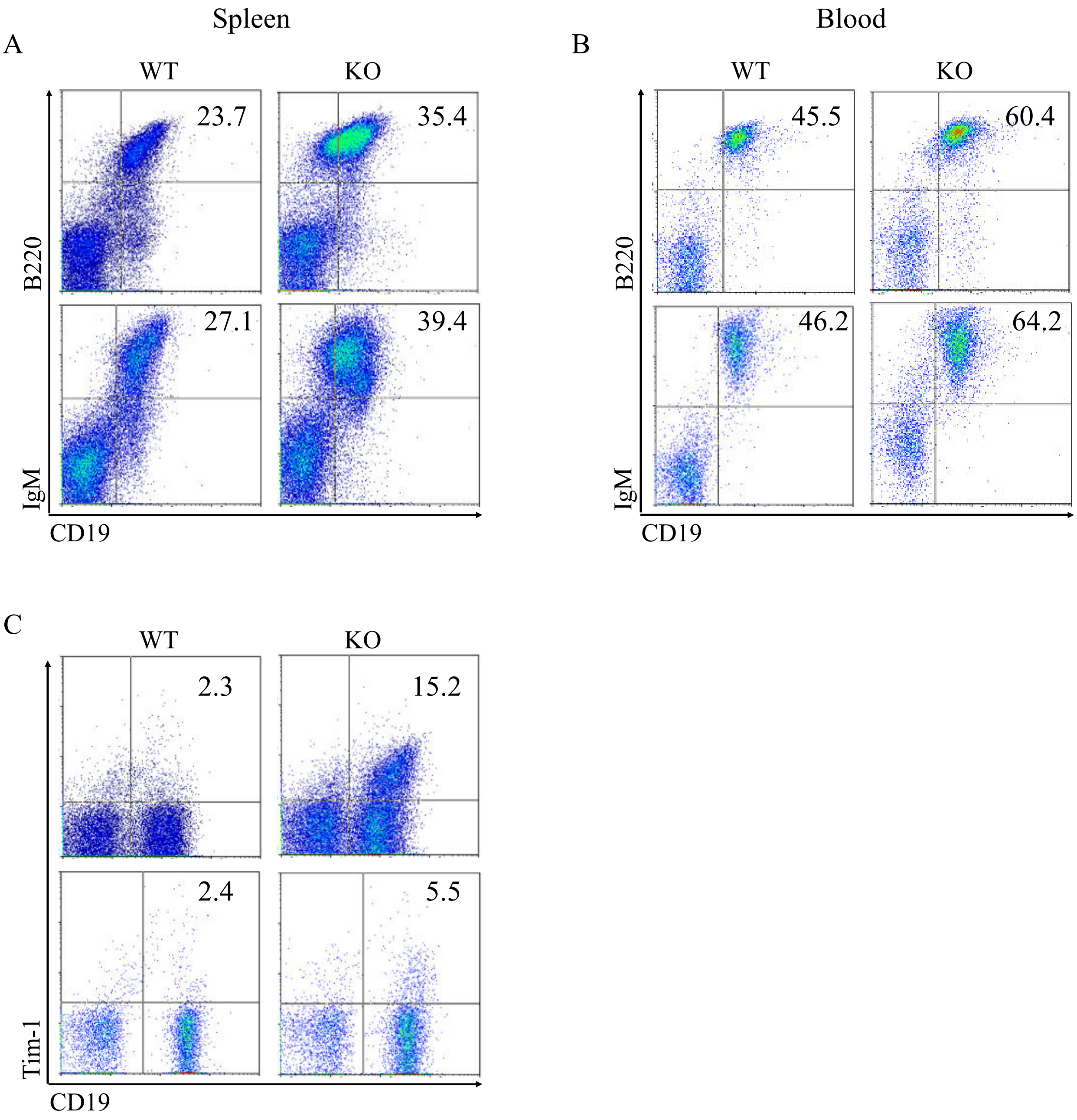

Supplementary figure 1. Representative B cell leukemia and CD19+ Tim-1+ cells in aged miR-15a/16 knockout mice (17 months).

Supplementary Figure

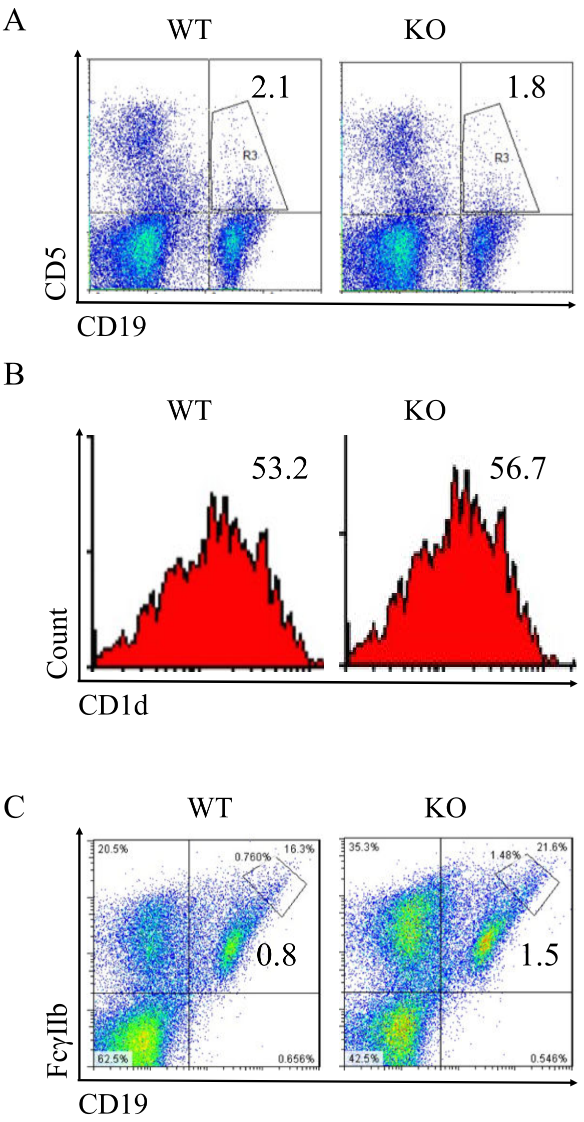

Supplementary figure 2. Representative results of splenic CD19^{+} CD5^{+} CD1d^{high} and CD19^{+} FcγIIb^{+} cells detected by flow-cytometry in the young miR-15a/16 knockout mice transplanted with H-22 cancer cells.

Supplementary Figure

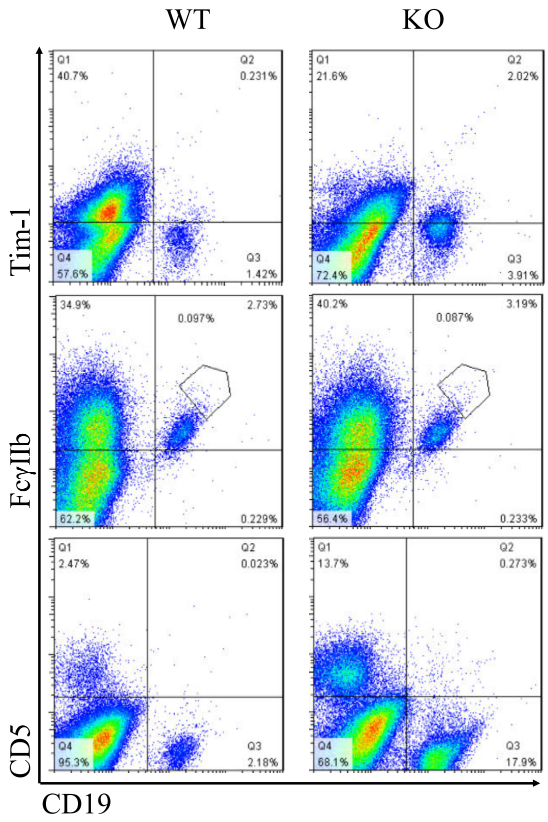

Supplementary figure 3. Representative results of tumor-derived $CD19^{+}$ Tim^{+}, $CD19^{+}$ $Fc\gamma IIb^{+}$, and $CD19^{+}$ $CD5^{+}$ cells detected by flow-cytometry in the young miR-15a/16 knockout mice transplanted with H-22 cancer cells.
